# Beantwortung epidemiologisch-rheumatologischer Fragestellungen durch Kooperation mit der bevölkerungsbasierten SHIP-Kohorte – Erkenntnisse für die Diagnostik der axialen Spondyloarthritis (axSpA)

**DOI:** 10.1007/s00393-021-01050-y

**Published:** 2021-07-15

**Authors:** J. Braun, A. Richter, C. Schmidt, X. Baraliakos

**Affiliations:** 1 Rheumazentrum Ruhrgebiet Herne, Ruhr-Universität Bochum, Claudiusstr. 45, 44649 Herne, Deutschland; 2 Institut für Community Medicine, Universitätsmedizin Greifswald, Greifswald, Deutschland

**Keywords:** Knochenmarködeme, Sakroiliitis, HLA B27, Ostitis condensans, Magnetresonanztomographie, Bone marrow edema, Sacroiliitis, HLA B27, Osteitis condensans, Magnetic resonance imaging

## Abstract

In diesem Artikel wird dargestellt, wie sich Fragestellungen hinsichtlich der rheumatischen Erkrankung axiale Spondyloarthritis (axSpA) in Zusammenhang mit der Verfügbarkeit neuer bildgebender Verfahren und neuer Medikamente über mehr als zwei Jahrzehnte in einer rheumatologischen Forschungsgruppe entwickelt haben. Insbesondere in den letzten Jahren ergaben sich durch die Kooperation mit der SHIP („Study of Health in Pomerania“)-Kohorte neue grundlegende Aspekte. Dabei bestand eine intensive Kooperation zwischen der Ruhr-Universität Bochum (Rheumazentrum Ruhrgebiet) und der Universitätsmedizin Greifswald (Forschungsverbund „Community Medicine“). Das Design der SHIP-Kohorte ist schon vor 10 Jahren veröffentlicht worden und der Kohortenansatz wurde im Bundesgesundheitsblatt dargestellt, wobei zentrale methodische Fragen ausführlich erörtert wurden. Im Jahr 2014 wurde ein Kooperationsprojekt des Rheumazentrums Ruhrgebiet/Ruhr-Universität Bochum mit der Abteilung Klinisch-Epidemiologische Forschung (KEF) von SHIP vereinbart, aus dem bereits interessante Ergebnisse hochrangig publiziert wurden. Um das Potenzial solcher Kooperationen zu betonen, werden wesentliche Inhalte mit Fokus auf die Magnetresonanztomographie (MRT) im Folgenden, auch unter historischen Aspekten, dargestellt.

## Abstract (en)

This article presents how, based on the availability of new imaging methods and medications, objectives regarding the rheumatic disease axial spondyloarthritis (axSpA) have developed over the course of more than two decades into a rheumatologic research group. During recent years, cooperation with the Study of Health in Pomerania (SHIP) cohort has given rise to new fundamental aspects. This involved intensive cooperation between the Ruhr University Bochum (*Rheumazentrum Ruhrgebiet*) and the Greifswald University Hospital (Community Medicine research collective). The design of the SHIP cohort was published 10 years ago and the cohort approach presented in the *Bundesgesundheitsblatt*, which also described central methodologic questions in detail. In 2014, a cooperation project between the Ruhr Rheumatology Center/Ruhr University Bochum and the SHIP Department of Clinical and Epidemiologic Research (*Klinisch-Epidemiologische Forschung*, KEF; SHIP-KEF) was established, which has already resulted in publication of interesting results in high-ranking journals. In order to stress the potential of such corporations, important contents thereof are presented herein, with a focus on MRI and consideration of historical aspects.

## Das SHIP-Projekt

Eine wesentliche Motivation zum Start des SHIP-Projekts [1] war es, die Prävalenz und Inzidenz häufiger, gesundheitsrelevanter subklinischer und klinischer Endpunkte (Outcomes) sowie von Risikofaktoren zu ermitteln, um die relativ niedrige Lebenserwartung in der Region erklären zu können [2]. Entsprechend zielt SHIP nicht auf eine bestimmte Erkrankung oder Erkrankungsgruppe, sondern versucht, die Begriffe „Gesundheit“ und „Krankheit“ mit einem möglichst breiten Ansatz zu erfassen. Daher werden longitudinal Daten zu möglichst vielen populationsrelevanten Risikofaktoren und Erkrankungen gesammelt. Das SHIP-Projekt wird in der Region Vorpommern, einschließlich der Landkreise Ost- und Nordvorpommern und der kreisfreien Städte Greifswald und Stralsund, ohne die Inseln Usedom und Rügen, durchgeführt und umfasst zwei voneinander unabhängige Kohorten. Zwischen 1997 und 2001 wurden 4308 (2193 Frauen) Erwachsene in der Basiserhebung (SHIP-0) der ersten Kohorte untersucht. Die Stichprobenziehung für SHIP erfolgte in Anlehnung an die MONICA/KORA-Studie nach einer zweistufigen stratifizierten Cluster-Methode [3]. Die Nettostichprobe umfasste 6265 Personen im Alter von 20 bis 79 Jahren; der Einladung zur Basisuntersuchung folgten mehr als zwei Drittel der Probanden (Rückmeldung 68,8 %). Folgeuntersuchungen fanden nach etwa 5 Jahren von 2002 und 2006 (SHIP‑1, *n* = 3300), danach wieder zwischen 2008 und 2012 (SHIP‑2, *n* = 2333) und von 2014 bis 2016 (SHIP‑3, *n* = 1718) statt. Von 2019 bis 2021 wird, nach über 20 Jahren Beobachtungszeit, die vierte Folgeerhebung dieser Kohorte durchgeführt. Parallel zu SHIP wurden 2008 weitere 10.000 Erwachsene der Region für die Basisuntersuchung einer zweiten Kohorte (SHIP-TREND-0) eingeladen, von denen 4420 Probanden bis 2012 teilnahmen. Die erste Nachuntersuchung (SHIP-TREND‑1, *n* = 2507) erfolgte zwischen 2016 und 2019. Für SHIP-TREND wurde eine alters- und geschlechtsstratifizierte, aber ansonsten randomisierte Stichprobe aus den inzwischen zentralisierten Registerdaten Mecklenburg-Vorpommerns gezogen.

In beiden Kohorten werden weitere Informationen zu inzidenten Ereignissen im Rahmen der Mortalitäts- und Morbiditäts-Follow-ups systematisch erhoben. Schon in der Konzeptionsphase wurden hohe Qualitätsstandards zur Durchführung von SHIP etabliert [3]. Insbesondere SHIP‑2 und SHIP-Trend beinhalteten ein Untersuchungsprogramm in einem Umfang, der zuvor noch nie in einer Bevölkerungsstudie angewendet wurde [1]. So ist SHIP die erste Bevölkerungsstudie, in die Ganzkörper-Magnetresonanztomographische (MRT)-Untersuchungen integriert wurden [4]. Diese MRT-Untersuchungen beinhalteten eine native Ganzkörper- sowie eine kontrastmittelgestützte Untersuchung an einem 1,5 T-Scanner. Das Projekt SHIP wird durch einen externen wissenschaftlichen Beirat begleitet.

## Bedeutung der Magnetresonanztomographie für die axiale Spondyloarthritis

Die SHIP-MRT-Untersuchungen auf Bevölkerungsebene waren für uns von großem Interesse, weil wir uns schon seit Langem mit dem Einsatz von MRT bei entzündlich-rheumatischen Erkrankungen des Achsenskeletts befassen – wie z. B. bei der axialen Spondyloarthritis (axSpA) – ein Terminus, der die klassische ankylosierende Spondylitis [5] inzwischen weitgehend abgelöst hat [6]. Die MRT ist auf dem besten Wege, die klassische Röntgendiagnostik bei axSpA abzulösen – mit Ausnahme der Diagnostik der Knochenneubildung, da Syndesmophyten und Ankylosen im konventionellen Röntgenbild nach wie vor besser zu sehen sind.

## Biopsien aus dem Sakroiliakalgelenk

Nach Publikation der ersten Erkenntnisse zum Stellenwert der MRT für die Diagnostik der akuten Sakroiliitis [7] war der nächste Schritt die Analyse von Biopsaten aus dem Sakroiliakalgelenk (SIG), die mithilfe von computertomographiegestützten Techniken (Prof. Bollow, damals auch UKBF Berlin) in Kombination mit einer Glukokortikoidinjektion gewonnen wurden [8]. Weitere Studien mit Biopsaten aus dem SIG folgten [9–13]. Die anfänglichen Versuche, Entzündung auch in der Wirbelsäule darzustellen, waren zunächst nur begrenzt erfolgreich [14, 15]. Eine detaillierte Studie zeigte, wo im SIG die meisten Veränderungen auftreten [16] und ein sehr früher Fallbericht zeigte, wo im SIG die Entzündung beginnt [17].

## Einführung der Biologika in die Therapie der axialen Spondyloarthritis

Basierend auf den Biopsieergebnissen, wurde 1999 [8] die erste Pilotstudie mit dem TNFα-Inhibitor Infliximab in Berlin durchgeführt [18], bald gefolgt von einer von uns geplanten und durchgeführten nationalen multizentrischen, randomisierten kontrollierten Studie [19], die die Grundlage für die EMA-Zulassung 2003 auf Basis von „unmet need“ bildete.

## Quantifizierung der Wirbelsäulenentzündung von Patienten mit axialer Spondyloarthritis

Parallel erfolgte die Entwicklung eines quantitativen Scoringsystems für Wirbelsäulenentzündung bei Patienten mit axSpA [20, 21], welches die Grundlage für die Bewertung von MRTs in vielen weiteren Studien darstellte [22–27] und sich dann auch als prognostisch bedeutsam für das Ansprechen auf entzündungshemmende Therapien erwies [28, 29]. Darüber hinaus wurden die bevorzugten Lokalisationen in der Wirbelsäule [30] und der Zusammenhang dieser Befunde mit konventionellen klinischen Messparametern untersucht [31] sowie technische Verbesserungen wie z. B. die VIBE-Technik aufgezeigt [32].

Ein wichtiger Meilenstein war der erste Nachweis des direkten Zusammenhangs zwischen Entzündung und Knochenneubildung [33], dem weitere detaillierte Hinweise zur Bedeutung fetthaltiger Veränderungen folgten [34]. Im Gegensatz dazu ist die Bedeutung der ebenfalls vorhandenen osteodestruktiven Veränderungen (Erosionen) in der Wirbelsäule noch unklar [35, 36]; das ist in den SIG sicher anders [32].

Durch die Klassifikationskriterien der Axial Spondyloarthritis International Society (ASAS) von 2009 ist die MRT neben dem Röntgen ein etablierter Teil der Diagnostik bei Verdacht auf axSpA [37]. Auch im Zusammenhang mit Überweisungsstrategien hat die MRT große Bedeutung [38]. Arbeitsgruppen der ASAS haben, auch in Kooperation mit OMERACT [39, 40], verschiedentlich Definitionen der MRT-Veränderungen in den SIG [41, 42] und der Wirbelsäule [40] publiziert, die vor allem für klinische Studien und Vergleiche in der Forschung relevant sind. Vor Kurzem ist das auch für die individuelle klinische Diagnostik klarer geworden [43]. Es steht zu erwarten, dass die MRT das konventionelle Röntgen in der Erstdiagnostik auch im Hinblick auf strukturelle Veränderungen ablösen wird; hinsichtlich der akuten Entzündung ist dies bereits länger bekannt. Die Wichtigkeit einer sorgfältigen Differentialdiagnose wurde in den letzten Jahren mehrfach betont [44, 45].

## Auswertung magnetresonanztomographischer Untersuchungen in der SHIP-Kohorte

Im Rahmen der Kooperation des Rheumazentrums Ruhrgebiet/Ruhr-Universität Bochum mit dem SHIP-Projekt wurden insgesamt die MRTs von 793 Probanden (49,4 % Männer, Durchschnittsalter 37,3 ± 6,3 Jahre, 8,4 % HLA B27+) im Alter von <45 Jahren ausgewertet, um die Häufigkeit von Knochenmarködemen (KMÖ) und fetthaltigen Veränderungen (FL) in SIG und Wirbelsäule (nur KMÖ), die denen bei axSpA ähneln, in einer Bevölkerungsstichprobe zu untersuchen [46, 47].

Dadurch bestand die Möglichkeit, die Wirbelsäule (sagittale Schnittführung, T1/T2-Sequenz) und SIG (semikoronare Schnittführung, „short tau inversion recovery“, STIR-Sequenz) in einer großen Stichprobe gezielt zu scoren. Dies wurde durch 2 Reader umgesetzt, welche die MRT-Bilder unabhängig voneinander auswerteten, um KMÖ in den SIG und den vertebralen Ecken (VE) zusammen mit FL nach den ASAS-Definitionen zu identifizieren. Dabei [46] wurde ≥1 KMÖ-Läsion bei 136 Probanden in den SIG (17,2 %) und bei 218 in der Wirbelsäule (27,5 %) gesehen, während bei 645 Probanden (81,4 %) ≥1 FL in der Wirbelsäule vorhanden waren. Die Untersuchung des Ausmaßes der KMÖ in den SIG zeigte aber, dass zwar häufig ≥1 Läsion betroffen war, jedoch ≥3 Läsionen nur in 8 Fällen vorkamen (1 %). In der Wirbelsäule war das ähnlich, wo KMÖ ≥3 bzw. ≥5 Läsionen bei 38 (4,8 %) bzw. nur 6 Probanden (0,8 %) vorkamen. Verglichen dazu kamen FL in der Wirbelsäule deutlich häufiger vor, mit ≥3 bzw. ≥5 Läsionen bei 351 (44,3 %) bzw. 185 (23,3 %) Probanden. Die logistische Regressionsanalyse zeigte, dass KMÖ und FL in VE mit höherem Alter pro Dekadenanstieg zusammenhing: OR 1,33 (95 % CI 1,02–1,72) und OR 1,73 (95 % CI 1,32–2,27).

In SHIP wurden entzündliche und fetthaltige MRT-Läsionen, die auf axSpA hindeuten können, vor allem in der Wirbelsäule, aber auch in den SIG gefunden. Dies deutet darauf hin, dass geringe MRT-Befunde in den SIG und der Wirbelsäule für die Diagnose und Klassifikation von axSpA keine große Rolle spielen. Die zunehmende Häufigkeit mit dem Alter legt nahe, dass mechanische Faktoren zumindest eine zusätzliche Rolle in der Pathogenese solcher MRT-Veränderungen spielen.

In einer darauf aufbauenden Arbeit [47] wurden mögliche Assoziationen zwischen klinischen Faktoren und dem Vorhandensein bzw. der Ausdehnung von KMÖ mittels logistischer bzw. negativer binomialer Regression analysiert. Darüber hinaus wurden Verknüpfungen mit Datensätzen von Krankenkassenleistungsdaten hergestellt, um Teilnehmer mit axSpA zu identifizieren. In den MRTs von 793 Probanden, darunter 401 Frauen (50,6 %) war das Vorhandensein von KMÖ in den SIG stark mit einer Entbindung im letzten Jahr assoziiert (4,47 OR: 1,49–13,41). Für das Ausmaß des KMÖ in den SIG wurde ebenfalls eine starke Assoziation mit einer Entbindung im letzten Jahr ermittelt (Inzidenzquotient: 4,52 [95 % CI 1,48–13,84]). Auch die Assoziation mit HLA B27 war deutlich, mit 2,32 (95 % CI 1,3–4,14) ebenso wie der Body-Mass-Index (BMI 25–30 vs. <25 kg/m^2^) mit 1,86 (95 % CI 1,19–2,89) und dem Vorkommen von Rückenschmerzen in den letzten 3 Monaten mit 1,55 (95 % CI 1,04–2,31), während für KMÖ in der Wirbelsäule Assoziationen mit dem Alter pro Dekade mit 1,46 (95 % CI 1,13–1,90) und körperlich anstrengender Arbeit mit 1,46 (95 % CI 1,06–2,00) vorlagen [47]. Dabei hatten unter den 694 (87,5 %) Teilnehmern, für die eine Datensatzverknüpfung mit Kassendaten möglich war, nur 9 (1,3 %) eine Eintragung für axSpA (ICD M45.09) – was in etwa der erwarteten Prävalenz von axSpA entspricht [48].

Diese Daten unterstützen die Hypothese einer mechanischen Belastung, z. B. durch Entbindung, die zu KMÖ in den SIG der Allgemeinbevölkerung im Alter von <45 Jahren führt. Die Rolle eines positiven HLA-B27-Allelstatus in der Pathogenese von axSpA wird durch diese Daten eher als ein Faktor für den Schweregrad als für Anfälligkeit für KMÖ in den SIG gestützt, was unseren vor Kurzem formulierten Vorstellungen zur Rolle dieses MHC-Klasse-I-Moleküls und prominenten genetischen Faktors entspricht [49].

## Schlussfolgerung

Diese Entwicklung zeigt zum einen, wie die konsequente Verfolgung von Forschungszielen zu klinisch relevanten Ergebnissen führen kann und zum anderen, dass die interdisziplinäre Kooperation zwischen Universitäten, einzelnen Abteilungen und auch anderen wissenschaftlichen Einrichtungen erheblich zu Erfolgen beitragen kann.

Über viele Jahre konnte die Bedeutung der MRT für die Diagnostik der axialen Spondyloarthritis immer klarer gemacht werden, wobei zuletzt vor allem die Feststellung der Häufigkeit von Knochenmarködemen im Achsenskelett in der Allgemeinbevölkerung sehr wichtig war – zum einen, weil es zu wesentlichen diagnostischen Hinweisen geführt hat und zum anderen, weil sich bei der Analyse von Einflussfaktoren Anhaltspunkte für mechanische Ursachen – wie Osteitis condensans – bei Frauen ergaben. Darüber hinaus ergaben sich weitere Anhaltspunkte für wesentliche Unterschiede zwischen Männern und Frauen hinsichtlich der Pathogenese der axialen Spondyloarthritis, der sich zum einen auf HLA B27 bezieht und zum anderen eine Rolle für das Körpergewicht von Frauen suggeriert. Noch unveröffentlichte Untersuchungsergebnisse deuten zudem darauf hin, dass hinsichtlich der genetischen Prädisposition durch HLA B27 Unterschiede zwischen Männern und Frauen bestehen, die noch weiter geklärt werden müssen.
